# Association Between Serum Iron Status and Muscle Mass in Adults: Results From NHANES 2015–2018

**DOI:** 10.3389/fnut.2022.941093

**Published:** 2022-07-11

**Authors:** Zhi Chen, Jing Chen, Chenyang Song, Jun Sun, Wenge Liu

**Affiliations:** ^1^Department of Orthopedic Surgery, Fujian Medical University Union Hospital, Fuzhou, China; ^2^Department of Ophthalmology, Fujian Provincial Hospital, Fuzhou, China; ^3^Department of Emergency, Zhaotong Traditional Chinese Medicine Hospital, Zhaotong, China

**Keywords:** ferritin, iron, skeletal muscle, NHANES, sarcopenia

## Abstract

**Background:**

Iron deficiency or overload may contribute to complications associated with diseases, but the link between iron status and skeletal muscle disorder is poorly understood. This study aimed to investigate the relationship between serum iron status, reflected by serum ferritin concentration, and muscle mass in U.S. adults.

**Methods:**

We utilized data from National Health and Nutrition Examination Survey (NHANES) 2015-2018 for analysis. Data on serum ferritin, appendicular skeletal muscle mass (ASM), body mass index (BMI) and confounding factors were extracted and analyzed. Multivariate linear regression analyses and smooth curve fittings were employed to investigate the association between serum ferritin and muscle mass. Subgroup analysis based on iron status, age, gender and race were performed.

**Results:**

A total of 2,078 participants were included, and divided into iron deficiency (*n* = 225), normal iron status (*n* = 1,366), and iron overload (*n* = 487) groups. Participants with iron overload had significantly lower ASM and appendicular skeletal muscle index (ASMI) (ASM: 19.329 ± 4.879, ASMI: 0.709 ± 0.138) compared to those with iron deficiency (ASM: 22.660 ± 6.789, ASMI: 0.803 ± 0.206) and normal iron status (ASM: 22.235 ± 6.167, ASMI: 0.807 ± 0.201). The serum ferritin was negatively linked with muscle mass after adjusting for potential confounders (β = −0.0001, 95% CI: −0.0001, −0.0000). When stratified by iron status, the trend test between them remained significant (P for trend: 0.008). Furthermore, subgroup analysis identified a stronger association in men (β = −0.0001, 95% CI: −0.0002, −0.0001), age ≥ 40 years (β = −0.0001, 95% CI: −0.0002, −0.0000), non-Hispanic black (β = −0.0002, 95% CI: −0.0003, −0.0001) and other races (β = −0.0002, 95% CI: −0.0003, −0.0000).

**Conclusions:**

Our study revealed an inverse relationship between serum iron status and muscle mass in adults. This finding improves our understanding of the impact of serum iron status on muscle mass, and sheds new light on the prevention and treatment of muscle loss.

## Introduction

Aging is a progressive, deleterious process that adversely affects the function of multiple organ systems (1). The effects of aging in skeletal muscle have been characterized by decline of muscle mass, strength and function, which is commonly referred to as sarcopenia (2). It is well accepted that sarcopenia is closely related with adverse outcomes, such as falls, fractures, disability, frailty, and mortality (2, 3).

Although the epidemiology and clinical manifestations of sarcopenia are much better understood, its pathogenesis has not been fully elucidated (4). Recently, the role of minerals in muscle metabolism has generated much attention. A number of studies have confirmed the relationships between magnesium, selenium, calcium and skeletal muscle disorder (5–7). Iron is an essential trace element, which plays a pivotal role in cellular metabolism, survival and proliferation (8). Several studies reported that iron deficient caused anemia, neurocognitive dysfunction, and impaired functional capacity (9, 10), whereas iron overload resulted in osteoporosis, neurodegeneration, and cardiovascular diseases (11–13). In the field of skeletal muscle research, recent studies also demonstrated an association between iron status and muscle disorders in experimental animals (14, 15), but the link between serum iron status and muscle mass in human is poorly understood. Therefore, we performed this study to investigate the relationship between serum iron status, reflected by serum ferritin concentration, and muscle mass in U.S. adults.

## Materials and Methods

### Data Source

The National Health and Nutrition Examination Survey (NHANES) is a cross-sectional survey, which provides a large amount of information on the nutrition and health of the non-institutionalized U.S. population. The NHANES protocols are approved by the Ethic Review Board of the National Center for Health Statistics, and all participants have consented to the use of their information for research (16).

### Study Population

**Figure 1:**
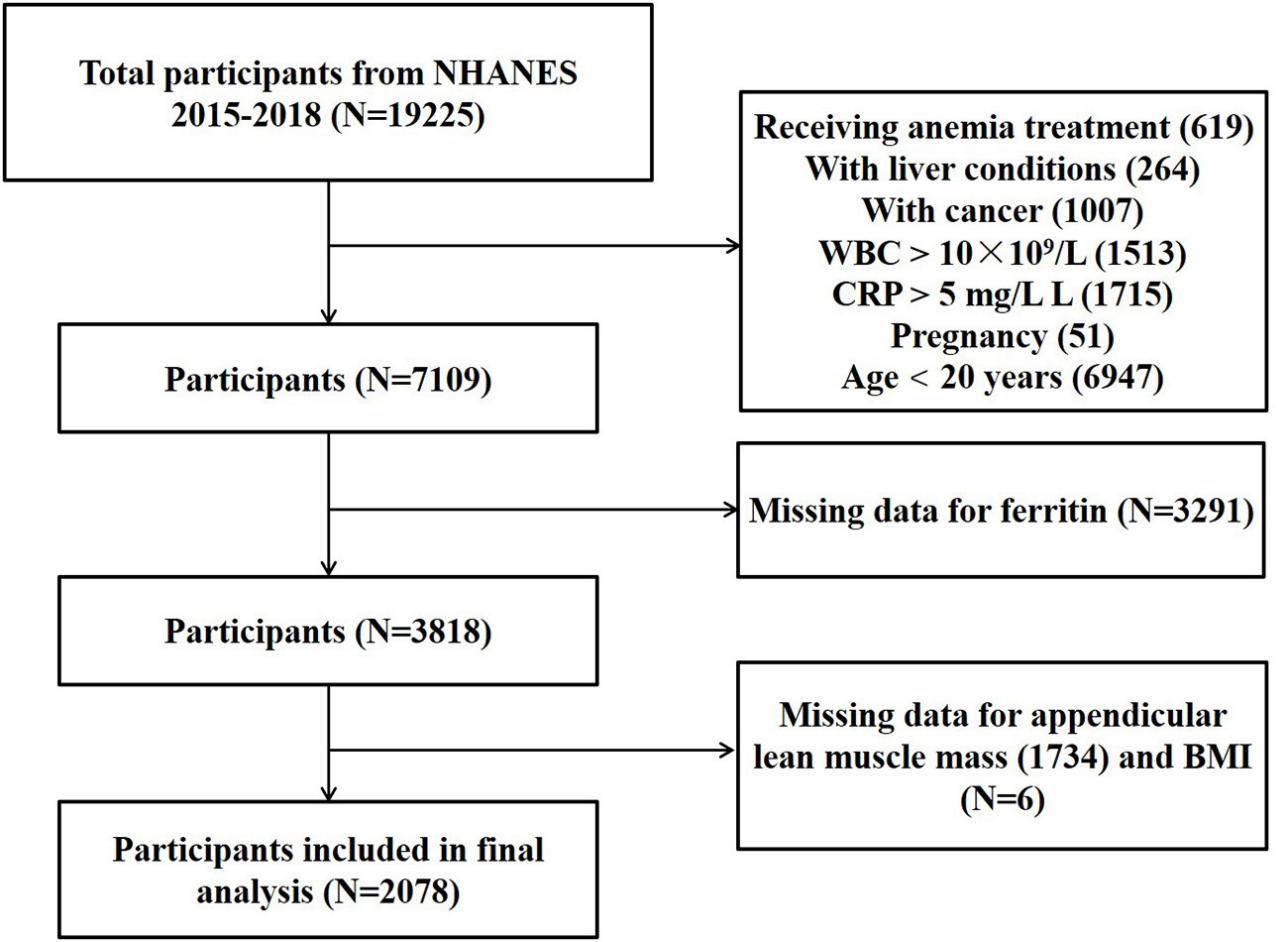
The participant selection flow-chart.

For this study, we utilized publicly available data from 2 two-year cycles (2015–2016 and 2017–2018) of NHANES for analysis. The inclusion criteria were as follows: participants aged ≥20 years old, with complete data of ferritin, ASM and BMI, without factors (inflammation, infection, malignancy, etc.) that might influence serum iron status. We excluded participants who received anemia treatment (*n* = 619), with a liver condition (*n* = 264), a malignancy (*n* = 1,007), infection (white blood cell count >10 × 10^9^/L; *n* = 1,513), inflammation (CRP > 5 mg/L; *n* = 1,715), pregnant (*n* = 51), age <20 years old (*n* = 6,947), with missing data of ferritin (*n* = 3,291), ASM (*n* = 1,734) and BMI (*n* = 6). (The participant selection flow-chart is demonstrated in Figure 1). For each included participants, data on ferritin, ASM, BMI and confounding factors were extracted and aggregated.

### Study Variables

#### Muscle Mass

The lean soft tissue measurement was conducted using whole-body dual-energy x-ray absorptiometry (DXA) scans (Hologic, Inc., Bedford, Massachusetts). ASM (g) was calculated as the sum of lean soft tissue mass of both arms and legs. BMI (kg/m^2^) was defined as weight divided by height^2^. We further quantified muscle mass using ASMI, calculated as the ASM divided by BMI (17).

### Serum Ferritin

The serum ferritin (ug/L) was measured using electrochemiluminescence immunoassay method. Serum iron status was classified according to WHO guideline: males and females with serum ferritin <15 ug/L were defined as iron deficiency; females with serum ferritin >150 ug/L and males with serum ferritin >200 ug/L were defined as iron overload; other serum ferritin levels were considered as normal iron status (18).

### Other Covariates

According to literatures, the following variables were regarded as covariates. Demographic variables including age, sex (Male, Female), race (Hispanic, Non-Hispanic White, Non-Hispanic Black, and Other Races), education (< High school, High school, >High school) were collected from demographic data. The medical conditions including hypertension and diabetes were defined by the participants' self-reported physician's diagnosis. The serum albumin (g/dL), globulin (g/dL), AST (IU/L), total protein (g/dL), uric acid (mg/dL) were measured using Beckman UniCel^®^ DxC 800 Synchron & Beckman UniCel^®^ DxC 660i Synchron Access Clinical Systems (Identical Method). White blood cell counts (1,000 cells/uL) and hemoglobin (g/dL) measurement were performed using the Coulter^®^ DxH 800 analyzer. High sensitivity C-Reactive Protein (HS-CRP) was measured using Beckman UniCel^®^ DxC 600 Synchron & Beckman UniCel^®^ DxC 660i Synchron Access Clinical Systems (Identical Method). Details of the specimen collection, processing, quality assurance and monitoring are available at NHANES website.

### Statistical Analysis

All analyses were performed using R 3.4.3 (https://www.r-project.org/) and EmpowerStats software (http://www.empowerstats.com), with *P* < 0.05 considered to be statistically significant. Sample weights were taken into account for calculating all estimates. Weighted linear regression models (for continuous variables) and weighted chi-square tests (for categorical variables) were performed to compare the baseline characteristics. Weighted multivariable linear regression analysis was performed to estimate the relationship between serum ferritin and ASMI. Subgroup analysis stratified by iron status, age, gender, and race were also performed. Furthermore, smooth curve fitting was applied to examine linear or non-linear relationship between them. If a non-linear relationship was identified, threshold effect analysis was conducted using two-piecewise linear regression model.

## Results

Based on the inclusion and exclusion criteria, a total of 2,078 participants were included for final analysis. As shown in Table 1, participants were categorized according to their iron status. There were 225 participants with iron deficiency, 1,366 participants with normal iron status, 487 participants with iron overload. Participants with iron overload had significantly lower serum uric acid, hemoglobin, HS-CRP, ASM, ASMI, and higher white blood cell count and ferritin when compared to the other groups.

**Table 1:** Baseline characteristics of study participants.

	**Iron status**			
**Characteristics**	**Deficiency**	**Normal**	**Overload**	***P*** **-value**
*n*	225	1,366	487	
Age (year)	36.715 ± 11.054	37.957 ± 11.586	37.305 ± 10.883	0.23837
BMI (kg/m^2^)	27.903 ± 5.403	27.277 ± 5.962	27.371 ± 5.847	0.35004
Albumin (g/dL)	4.261 ± 0.286	4.248 ± 0.294	4.224 ± 0.322	0.21993
AST (IU/L)	22.849 ± 10.207	22.912 ± 13.495	21.424 ± 10.103	0.08039
Globulin (g/dL)	2.889 ± 0.331	2.912 ± 0.392	2.885 ± 0.394	0.34961
Total Protein (g/dL)	7.150 ± 0.365	7.160 ± 0.410	7.109 ± 0.394	0.05994
Uric acid (mg/dL)	5.042 ± 1.363	5.146 ± 1.333	4.628 ± 1.203	<0.00001
Hemoglobin (g/dL)	14.165 ± 1.405	14.231 ± 1.393	13.695 ± 1.267	<0.00001
WBC (1,000 cells/uL)	6.871 ± 1.438	6.683 ± 1.534	7.000 ± 1.567	0.00037
HS-CRP (mg/L)	2.663 ± 1.811	1.670 ± 1.069	0.677 ± 0.526	<0.00001
Ferritin (ug/L)	9.248 ± 3.278	70.830 ± 41.379	314.894 ±184.838	<0.00001
ASM (kg)	22.660 ± 6.789	22.235 ± 6.167	19.329 ± 4.879	<0.00001
ASMI	0.803 ± 0.206	0.807 ± 0.201	0.709 ± 0.138	<0.00001
Gender				<0.00001
Male	44.528	47.287	15.442	
Female	55.472	52.713	84.558	
Race				0.61370
Hispanic	24.056	19.935	19.207	
Non-Hispanic White	50.298	57.212	58.135	
Non-Hispanic black	11.761	10.602	11.000	
Other	13.885	12.251	11.659	
Education				0.28505
<High school	9.828	8.692	11.584	
High school	21.032	22.398	19.006	
>High school	69.140	68.910	69.410	
Hypertension				0.00129
Yes	22.856	18.652	12.520	
No	77.144	81.348	87.480	
Diabetes				0.01329
Yes	5.084	3.697	1.298	
No	94.916	96.303	98.702	

*BMI, Body mass index; AST, aspartate Aminotransferase; WBC, White blood cell count; HS-CRP, High-sensitive C-reactive protein; ASM, appendicular skeletal muscle mass; ASMI, Appendicular skeletal muscle index. Mean ± SD for continuous variables: P-value was calculated by weighted linear regression model. Percent for categorical variables: P-value was calculated by weighted chi-square test*.

### Association Between Serum Ferritin and Muscle Mass

Table 2 shows the results of the multivariate linear regression analysis. The serum ferritin was negatively linked with muscle mass in the unadjusted model (model 1: β = −0.0002, 95%CI: −0.0003, −0.0001). After adjusting for covariates, this significant association was remained apparent in the fully adjusted model (model 3: β = −0.0001, 95%CI: −0.0001, −0.0000), but not in partially adjusted model (model 2: β = −0.0000, 95%CI: −0.0000, 0.0000). Similarly, when stratified by iron status, the trend test between them remained significant in unadjusted model and fully adjusted model (P for trend <0.05).

**Table 2:** Relationship between ferritin and ASMI.

**Outcome**	**Model 1**		**Model 2**		**Model 3**	
	**β (95%CI)**	***P*** **-value**	**β (95%CI)**	***P*** **-value**	**β (95%CI)**	***P*** **-value**
Ferritin	−0.0002 (–0.0003,−0.0001)	<0.000001	−0.0000 (–0.0000, 0.0000)	<0.625492	−0.0001 (–0.0001, −0.0000)	0.005600
**Iron status**						
Deficiency	Reference		Reference		Reference	
Normal	0.0043 (–0.0230, 0.0316)	<0.755540	−0.0038 (–0.0207, 0.0131)	0.661284	−0.0192 (–0.0363, −0.0021)	0.027819
Overload	−0.0937 (–0.1244, −0.0630)	<0.000001	−0.0024 (–0.0217, 0.0168)	0.803185	−0.0307 (–0.0525, −0.0089)	0.005915
P for trend	<0.001		0.894		0.008	

*Model 1: no covariates were adjusted. Model 2: gender, age and race were adjusted. Model 3: gender, age, race, BMI, education, hypertension, diabetes, albumin, AST, Globulin, total protein, uric acid, white blood cell count, hemoglobin, and HS-CRP were adjusted*.

In subgroup analysis stratified by age, this negative association was maintained in participants ≥40 years old (model 1: β = −0.0002, 95%CI: −0.0003, −0.0002 and model 3: β = −0.0001, 95%CI: −0.0002, −0.0000), but not in participants <40 years old (model 2: β = 0.0000, 95%CI: −0.0000, 0.0001 and model 3: β = −0.0000, 95%CI: −0.0001, 0.0000). When stratified by gender, the significant association was maintained in men (all three models), but not in women (all three models). When stratified by race, significantly negative associations were observed only in Non-Hispanic Black and Other Races (all three models) (Table 3).

**Table 3:** Relationship between ferritin and ASMI.

**Outcome**	**Model 1**		**Model 2**		**Model 3**	
	**β (95%CI)**	***P*** **-value**	**β (95%CI)**	***P*** **-value**	**β (95%CI)**	***P*** **-value**
**Stratified by age**						
Age <40 years	−0.0002 (−0.0003, −0.0001)	0.00013	0.0000 (−0.0000, 0.0001)	0.452894	−0.0000 (−0.0001, 0.0000)	0.500286
Age ≥40 years	−0.0002 (−0.0003, −0.0002)	<0.000001	−0.0000 (−0.0001, 0.0000)	0.119202	−0.0001 (−0.0002, −0.0000)	0.001150
**Stratified by gender**						
Male	−0.0002 (−0.0003, −0.0001)	<0.000197	−0.0002 (−0.0003, −0.0001)	0.000220	−0.0001 (−0.0002, −0.0001)	0.000652
Female	0.0000 (−0.0000, 0.0001)	0.093336	0.0000 (−0.0000, 0.0001)	0.080967	0.0000 (−0.0000, 0.0001)	0.826401
**Stratified by race**						
Hispanic	−0.0001 (−0.0002, −0.0000)	0.007248	0.0000 (−0.0001, 0.001)	0.986537	−0.0000 (−0.0001, 0.0000)	0.316795
Non-Hispanic white	−0.0002 (−0.0003, 0.0001)	0.000734	0.0000 (−0.0000, 0.0001)	0.603048	−0.0000 (−0.0001, 0.0000)	0.347893
Non-Hispanic black	−0.0005 (−0.0007, −0.0003)	<0.000001	−0.0001 (−0.0002, −0.0000)	0.014483	−0.0002 (−0.0003, −0.0001)	0.000752
Other races	−0.0003 (−0.0004, −0.0002)	0.000007	−0.0001 (−0.0002, −0.0000)	0.044939	−0.0002 (−0.0003, −0.0000)	0.003603

*Model 1: no covariates were adjusted. Model 2: gender, age and race were adjusted. Model 3: gender, age, race, BMI, education, hypertension, diabetes, albumin, AST, Globulin, total protein, uric acid, white blood cell count, hemoglobin, and HS-CRP were adjusted*.

### Threshold Effect Analysis

**Figure 2:**
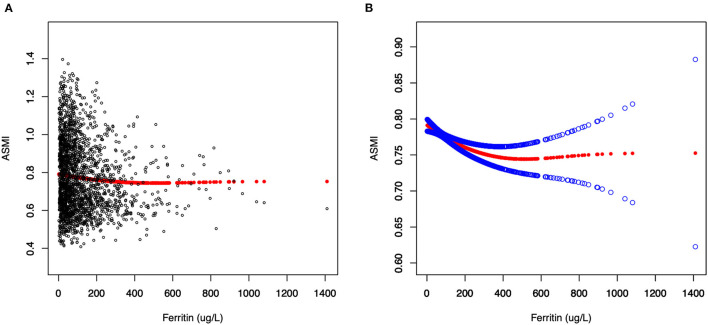
The relationship between serum ferritin and muscle mass. **(A)** Each black point represents a sample. **(B)** Solid red line represents the smooth curve fit between variables. Blue bands represent the 95% of confidence interval from the fit.

**Figure 3:**
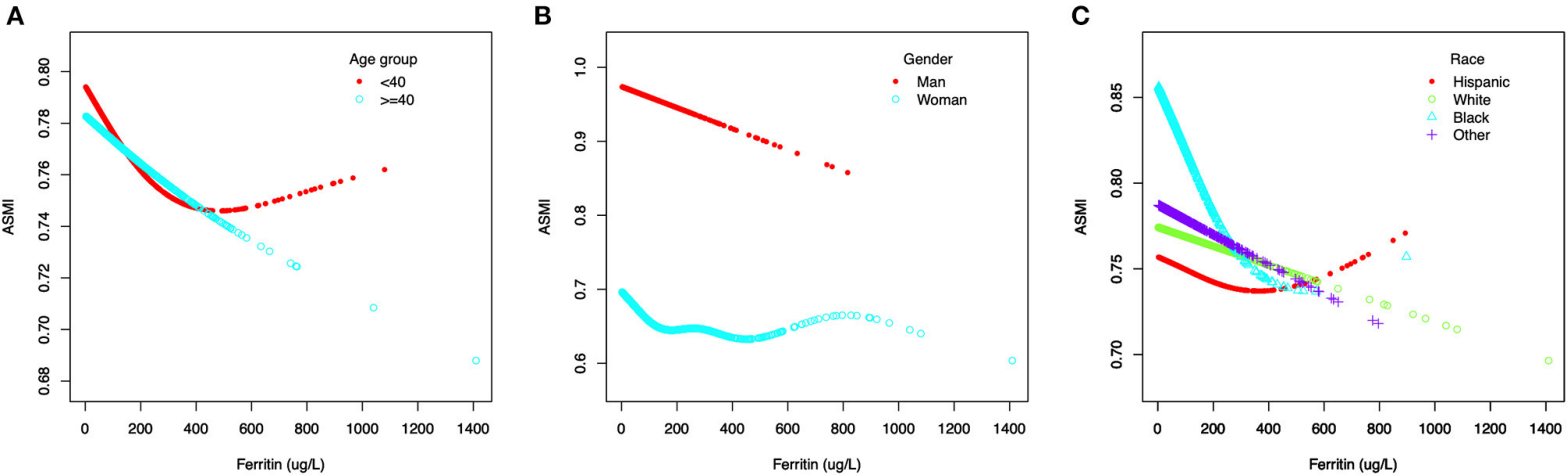
The association between serum ferritin and muscle mass, stratified by age **(A)**, stratified by gender **(B)**, stratified by race **(C)**.

Generalized additive models and smooth curve fittings used to detect the non-linear relationship between serum ferritin and muscle mass are shown in Figures 2, 3. The results demonstrated linear relationships between ferritin and muscle mass in participants ≥40 years old (Figure 3A) and men (Figure 3B), and non-linear relationships in participants <40 years old (Figure 3A), Hispanic and Non-Hispanic Black (Figure 3C). Among participants <40 years old and Hispanics, the relationship between ferritin and muscle mass was a U-shape curve, with the infection point identified at 231 ug/L in participants <40 years old and 223 ug/L in Hispanics (Table 4).

**Table 4:** Threshold effect analysis of ferritin on ASMI in age <40 years, Hispanic and Non-Hispanic Black using the two-piecewise linear regression model.

	**Adjusted β (95%CI)**	***P*** **-value**
**Age <40 years**		
Ferritin < 231	−0.0002 (−0.0003, −0.0001)	0.0038
Ferritin > 231	0.000 (−0.0001, 0.0001)	0.4125
**Hispanic**		
Ferritin < 223	−0.0002 (−0.0003, −0.0000)	0.0430
Ferritin > 223	0.0000 (−0.0001, 0.0002)	0.4421
Non-Hispanic Black		
Ferritin < 315	−0.0004 (−0.0005, −0.0002)	<0.0001
Ferritin > 315	0.0002 (−0.0002, 0.0005)	0.3581

*Gender, age, race, BMI, education, hypertension, diabetes, albumin, AST, Globulin, total protein, uric acid, white blood cell count, hemoglobin, and HS-CRP were adjusted*.

## Discussion

Muscle loss is the main feature of sarcopenia, which contributes to increased risk of fractures, impaired physical function, and reduced quality of life (19). Although remarkable strides have been made in understanding its etiology and pathogenesis, little is known about the relationship between serum iron status and muscle mass. The findings of our study demonstrated a significantly negative association between serum ferritin and muscle mass, even after adjusting for potential confounders. The association was stronger in men, age ≥40 years, non-Hispanic black and other races.

As an essential micronutrient, iron plays crucial roles in various biological processes, including enzymatic activities, mitochondrial function, energy metabolism, RNA and DNA synthesis (20, 21). However, due to a lack of effective excretory route, iron accumulation occurs easily during aging process (22). There is growing evidence that excess iron is significantly correlated with multiple diseases, including liver fibrosis, heart attack, neurodegeneration, and cancer (11, 23). In regarding to musculoskeletal system, several experimental studies also detected a link between iron accumulation and muscle disorders. Altun et al. (24) and Jung et al. (25) found a significantly elevated non-heme iron level in the skeletal muscle of aged rats. Xu et al. (26) indicated the iron accumulation might be considered as a hallmark of aged skeletal muscle. In another study, Reardon et al. demonstrated that intraperitoneal administration of iron to mice caused not only an increased oxidative stress within muscle tissue, but also a significantly decreased muscle force (15). Furthermore, Huang et al. (14) revealed that ferroptosis, an iron-dependent form of programmed cell death, triggered by iron accumulation might be a primary cause of muscle loss. Taken together, these experimental results strongly suggested that iron accumulation might be a potential contributor to the decline of muscle mass and function.

In contrast to experimental studies, the results of clinical studies remain controversial. While Bartali et al. (27) did not find a significant association between serum iron level and physical function in a longitudinal study involving 698 participants. Waters et al. (28) indicated a significant association between iron intake and gait speed in 315 older adults. Nakagawa et al. (29) reported that higher serum ferritin, even mildly, was significantly correlated in an inverse manner with lower muscle strength in 300 hemodialysis patients. In another cohort study involving 639 hospitalized elderly, Perna et al. (30) revealed a significantly higher serum ferritin level in sarcopenia group compared with the health controls. Furthermore, the serum ferritin levels exceeded the normal range (>145 ug/dl) in patients with sarcopenia (30). Our study enrolled a larger community-dwelling healthy participants, the result showed a significantly negative association between serum ferritin and muscle mass. Moreover, such association was influenced by gender, age, and ethnicity, with stronger associations among men, age ≥40 years, non-Hispanic black and other races.

Although the above experimental and clinical findings demonstrated a negative relationship between iron status and muscle mass, the underlying mechanisms remain to be fully elucidated. There is growing evidence that elevated reactive oxygen species (ROS) and increased local oxidative stress caused by excess iron may play an important role. In one hand, ROS can cause lipids, proteins, mitochondria, and nucleic acids damage, finally resulting in cellular dysfunction and ferroptosis (14, 24, 31). On the other hand, oxidative stress may lead to muscle tissue damage by increasing expression of inflammatory cytokines, such as interleukin-1 (IL-1), tumor necrosis factor-α (TNF-α), and IL-6 (32). Furthermore, several studies indicated the iron accumulation and insulin resistance were interconnected. It has been established that insulin resistance is closely linked to muscle loss (23, 33).

## Limitation

There were some limitations in our study. Firstly, due to the cross-sectional nature of this study, no causal relationship between serum ferritin and muscle loss could be concluded. Secondly, data on muscle strength and function were not available in this study, so the relationships between these variables and ferritin could not be investigated. Finally, whole body DXA scans were only performed to participants aged 8–59 years old in NHANES, there was a lack of research on elderly participants who were more likely to suffer from muscle loss. Therefore, more research and exploration in this field are still needed.

## Conclusion

The present study revealed an inverse relationship between serum iron status and muscle mass in community-dwelling adults, especially in men, age ≥40 years, non-Hispanic black and other races. This finding improves our understanding of the impact of serum iron status on muscle mass, and sheds new light on the prevention and treatment of muscle loss.

## Data Availability Statement

The original contributions presented in the study are included in the article/supplementary material, further inquiries can be directed to the corresponding author.

## Ethics Statement

The studies involving human participants were reviewed and approved by the National Center for Health Statistics Ethics Review Board. The patients/participants provided their written informed consent to participate in this study.

## Author Contributions

ZC wrote the manuscript. ZC, JC, CS, and JS collected and analyzed the data. WL designed this study and revised the manuscript. All authors read and approved the final manuscript.

## Conflict of Interest

The authors declare that the research was conducted in the absence of any commercial or financial relationships that could be construed as a potential conflict of interest.

## Publisher's Note

All claims expressed in this article are solely those of the authors and do not necessarily represent those of their affiliated organizations, or those of the publisher, the editors and the reviewers. Any product that may be evaluated in this article, or claim that may be made by its manufacturer, is not guaranteed or endorsed by the publisher.
